# A curated data resource to support safe carbon dioxide transport-route planning

**DOI:** 10.1016/j.dib.2023.109984

**Published:** 2023-12-17

**Authors:** Catherine Schooley, Lucy Romeo, Isabelle Pfander, Maneesh Sharma, Devin Justman, Jennifer Bauer, Kelly Rose

**Affiliations:** a National Energy Technology Laboratory Support Contractor, 1450 Queen Avenue SW, Albany, OR 97321, USA; b National Energy Technology Laboratory, 1450 Queen Avenue SW, Albany, OR 97321, USA; c National Energy Technology Laboratory Support Contractor, 3610 Collins Ferry Road, Morgantown, WV 26505, USA; d National Energy Technology Laboratory, 3610 Collins Ferry Road, Morgantown, WV 26505, USA

**Keywords:** Carbon capture and storage, CO_2_, Pipelines, Transport infrastructure, Decision support, Environmental justice, Social justice, Risk prevention

## Abstract

Supporting the national target of net-zero greenhouse gas emissions in the United States by 2050, the Bipartisan Infrastructure Law (BIL) authorized investments into carbon capture and storage (CCS), highlighting the need for the safe and sustainable transport of carbon dioxide (CO_2_). Curated to support CO_2_ pipeline route planning optimization and assess existing energy transport corridors, the CCS Pipeline Route Planning Database is a compilation of 47 publicly available, authoritative geospatial data resources, spanning the contiguous U.S., and some including Alaska and Hawaii. Key considerations were identified following comprehensive literature review, which included state legislation, known pipeline stressors, and energy, environmental, and social justice (EJSJ) considerations. Data layers were sorted into relevant categories (i.e., natural hazards, boundaries) and assigned preliminary weights representing potential social, environmental, and economic costs associated with routing pipelines. Version one of the CCS Pipeline Route Planning Database, made available on the Energy Data eXchange® (EDX), contains categorized vector features representing protected areas, public and energy infrastructure, EJSJ factors, potential risks, federal and state regulations and legislation, and natural features, along with associated metadata. This paper provides details on individual layers, methods used to identify data needs, acquire, and process the disparate data, as well as planned enhancements to future versions of this database.

Specifications Table

**Table:** 

Subject	Energy, sustainability, and the environment
Specific subject area	Carbon Capture and Storage Technology
Data format	Raw, Secondary data, Analyzed, Filtered, Geospatially processed
Type of data	Geospatial Data, Table, Figure
Data Collection	Following a literature review, data resources were identified and acquired based on their pertinence to carbon capture and storage (CCS) transport systems. Spatial data were collected from credible public resources, processed (ArcGIS Pro 3.0.3 and python v. 3.9), curated, and given preliminary weights based on existing pipeline construction recommendations and regulatory requirements. Data layers were also created using state-specific legislation. Only those datasets within the United States (U.S. and U.S. federal waters) were considered. Data acquired from online resources used standard configuration PC (Windows OS) and internet browser software (Microsoft Edge and Google Chrome).
Data source location	Primary data sources include U.S. Department of Energy, National Energy Technology Laboratory, U.S. Geological Survey, U.S. Census Bureau, U.S. Bureau of Land Management, U.S. Department of Transportation, National Register of Historic Places, National Park Service, Federal Emergency Management Agency, U.S. Environmental Protection Agency, Centers for Disease Control, Agency for Toxic Substances and Disease Registry, Oakridge National Laboratory, U.S. Energy Information Agency, National Oceanic and Atmospheric Administration, and U.S. Department of Agriculture
Data accessibility	Repository name: Energy Data eXchange® (EDX) Data Object Identifier (DOI) number: 10.18141/1965283 Direct URL to data: https://edx.netl.doe.gov/dataset/ccs-pipeline-route-planning-database-v1 The database and associated metadata are publicly available and can be downloaded from EDX using the link.

## 1 Value of the Data

• The CCS Pipeline Route Planning Database was developed to inform the acceleration of carbon management capabilities, supporting the U.S. Bipartisan Infrastructure Law's (BIL) aim of expanding CO_2_ transport infrastructure (i.e., pipelines) safely and responsibly to meet the goal of net-zero emissions by 2050.
• The CCS Pipeline Route Planning Database provides a one-stop-shop for 47 geospatial data layers spanning the U.S., which have been selected and processed to support CO_2_ transport-route planning.
• Version one of this resource contains spatial layers representing state and federal legislation, regulations, best-practices, natural hazards, as well as critical EJSJ factors.
• This spatial resource offers access to curated datasets, providing regulatory and government agencies, academic institutions, commercial and industrial operators, and public stakeholder groups with information to support CO_2_ transport-route planning and development.
• In addition, this digital database supports the development of new and existing tools and models for transport-route planning, providing data-driven insights into carbon capture, transport, and storage technologies to support environmentally and socially prudent planning.
• A valuable and growing resource for carbon capture, transport, and storage researchers and stakeholders, the CCS Pipeline Route Planning Database helps fill data gaps in current research and planning efforts, offering a critical resource for future research and provides novel datasets not previously available in a geospatial format.

## 2 Background

The BIL strives to enhance the U.S. ability for managing carbon by safely and conscientiously extending CO_2_ transportation infrastructure, such as pipelines. Developing complex energy transport systems require considerable time and monetary investments, underscoring the importance that all available and credible information is utilized during planning. Built to support these planning and development efforts, the CCS Pipeline Route Planning Database incorporates a variety of regulatory, topographic, and potential risk factors essential for the optimization of multi-modal route decision making. This geospatial database was also designed to support the development of CCS route planning tools and provide a curated compilation of credible resources representing critical factors, such as EJSJ considerations, public and energy infrastructure, and ground cover. Moreover, factors have been given preliminary weights to represent a measure of potential social, economic, and environmental costs associated with routing. The CCS Pipeline Route Planning Database provides a comprehensive, national, geospatial data resource to support actionable CCS science and accelerate the country's energy transition.

## 3 Data Description

Derived data sources representing various CO_2_ considerations were compiled into a geodatabase (Table 1). The geodatabase consists of six categories representing boundaries, CCS regulations by state, EJSJ considerations, hydrology, infrastructure, and natural hazards. Each category contains three to twelve spatial data layers.

**Table 1:** Geodatabase framework delineating datasets per category.

Category	Dataset
Boundaries	Protected Areas
	Wilderness Areas
	Tribally-Controlled Land
	National Monuments
	National Parks
	Historic Sites
	Urban or Developed Areas
	Weighted Land Cover
CCS by State	CO_2_ Atlas CCS Potential by State
	CO_2_ Taskforce Involvement by State
	CO_2_ Infrastructure Legislation Availability by State
Environmental, Energy, and Social Justice	National Risk Index by County
	National Risk Index Expected Annual Loss by County
	National Risk Index Social Vulnerability by County
	National Risk Index Community Resilience by County
	Environmental Justice Screen by Tract
	Social Vulnerability Index by Tract
	Environmental Justice Index by Tract
	Disadvantaged Communities
Hydrology	National Hydrology Dataset Wells
	National Hydrology Dataset Waterbodies
	National Hydrology Dataset Areas
	National Hydrology Dataset Flowlines
	Aquifers
	Groundwater Monitoring Locations
Infrastructure	Oil Gas Wells
	Natural Gas Pipeline Rights-of-Way
	Hydrocarbon Pipeline Rights-of-Way
	Areas Adjacent to Natural Gas Pipelines
	Areas Adjacent to Hydrocarbon Pipelines
	Primary Roads
	Secondary Roads
	Railroads
	Buildings
	Public Infrastructure
	Underground Structures
	Local Roads
Natural Hazards	National Risk Index Earthquake Risk by County
	National Risk Index Riverine Flood Risk by County
	National Risk Index Coastal Flood Risk by County
	National Risk Index Wildfire Risk by County
	National Risk Index Landslide Risk by County
	Soil Frost Action Potential
	Soil Steel Corrosion Potential
	Landslide Susceptibility
	Weighted Slope
	Floodplains

### 3.1 Boundaries

**Fig. 1:**
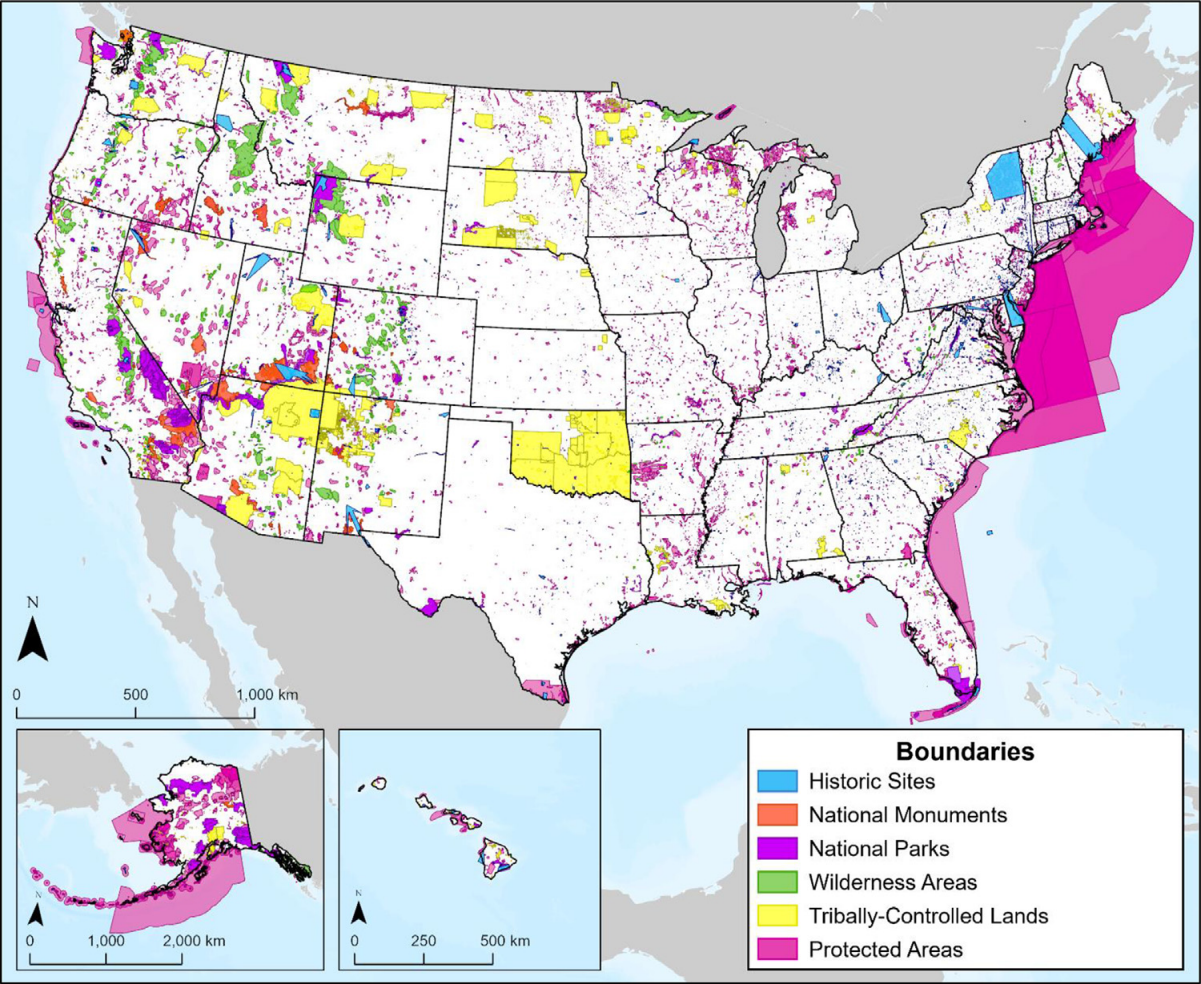
Map of boundary layers representing historic sites, national monuments, national parks, wilderness areas, tribally-controlled lands, and protected areas.

Acquired from various federal agencies, boundary layers represent areas of consideration for CCS pipeline route planning, as noted by federal or state legislation or existing pipeline regulations (Fig. 1). Many of the layers within this category are those that should be avoided for routing (i.e., national parks, tribally-controlled lands), while others contain a mix of areas that should be avoided or favored for routing considerations (i.e., land cover).

#### 3.1.1 Protected Areas

Protected areas represent terrestrial or marine spaces that were designated as having permanent protection from conversion of natural land cover and a mandated management plan in operation to maintain a natural state within which natural disturbance events may proceed without interference or are mimicked through management. These areas also include those that have mandated management plans in operation that maintain a primarily natural state but may receive uses or management practices that degrade the quality of existing natural communities, including suppression of natural disturbance (e.g., wildfires). Protected area boundaries were acquired from the Protected Areas Database [1], which was published by the U.S. Geological Survey's (USGS) Gap Analysis Project and includes information from managing agencies, as coordinated through the Federal Geographic Data Committee Federal Lands Working Group. The original data were filtered to represent areas considered classified by 49 CFR § 195 [3] as Unusually Sensitive Areas (USA), which might be impacted in the event of hazardous liquid pipeline release. Following past study findings, a setback distance of 91.44 m (300 ft) is included in this layer [2] (Fig. 1).

#### 3.1.2 Wilderness Areas

Wilderness areas are natural areas that have been designated as National Wilderness in the National Wilderness Preservation System through the 1964 Wilderness Act [4]. This federal designation provides a high level of protection by Congress. Once an area has been classified as a wilderness area, it must be managed to maintain or improve four significant wilderness qualities: untrammeled, natural, undeveloped, and provides opportunities for solitude or recreation. Wilderness area boundaries were acquired from the U.S. Department of Agriculture National Datasets service [1] (Fig. 1). Wilderness areas fall under 49 CFR § 195 [3] as USAs.

#### 3.1.3 Tribally-Controlled Lands

Tribally-controlled lands are areas that are owned by a tribe, tribal member, or by the U.S. government on behalf of a tribe or tribal member (Fig. 1). This includes federal tribal reservations which are lands reserved for a tribe or tribes held under treaty or other agreements with the U.S. These lands are not included under the definition of federal lands used in Mineral Leasing Act [5], which defines rules and regulations for rights-of-way (ROW) through federal lands. The Secretary of the Interior is authorized through 25 U.S. Code § 321 [6] to grant a ROW for the construction, operation, and maintenance of oil and gas pipelines through any lands that are tribally controlled. Acquired from the U.S. Census Bureau (USCB) [1] and included in this database, tribally-controlled lands also represent EJSJ considerations.

#### 3.1.4 National Monuments

National monuments represent public lands that have been deemed culturally, historically, or nationally significant (Fig. 1). National monuments are often determined by U.S. presidents through the authority granted to them by the 1906 Antiquities Act [7]. They may also be created through Congress. Many national monuments are owned by the National Park Service (NPS) and therefore excluded from the authorization provided by the Mineral Leasing Act for petroleum product pipelines on National Park lands. If not owned by the NPS, national monuments are subject to the rules and regulations of the Mineral Leasing Act pertaining to ROW through federal lands. National monument data were collected from the Bureau of Land Management National Surface Management Agency Areas database [1].

#### 3.1.5 National Parks

National parks are protected areas of land or water designated by Congress to conserve unique features like wildlife habitats and cultural sites for the benefit of current and future generations. National parks have rules for protection and can also be used for recreation, education, and research. National parks are excluded from the Mineral Leasing Act authorization that allows ROW for petroleum product pipelines through federal lands. These data were collected from the Bureau of Transportation Statistic's National Transportation Atlas Database [1] and filtered to represent areas owned and maintained by the NPS (Fig. 1).

#### 3.1.6 Historic Sites

Historic sites represent publicly available historic areas across the U.S. This layer represents locations with cultural or historical significance, identified and preserved by the NPS, which has maintained the National Register of Historic Places since the National Historic Preservation Act of 1966 [8]. Based on regulatory guidelines for reporting on cultural resource investigations for natural gas projects [9], historical sites should be avoided as much as possible, with a recommended minimum setback distance of 7.62 m (25 ft) from sites when using directional drilling. Originally acquired from the National Register [1], this layer includes historic buildings, districts, sites, and structures with a 7.62 m (25 ft) spatial setback around each historic site (Fig. 1).

#### 3.1.7 Urban or Developed Areas

**Fig. 2:**
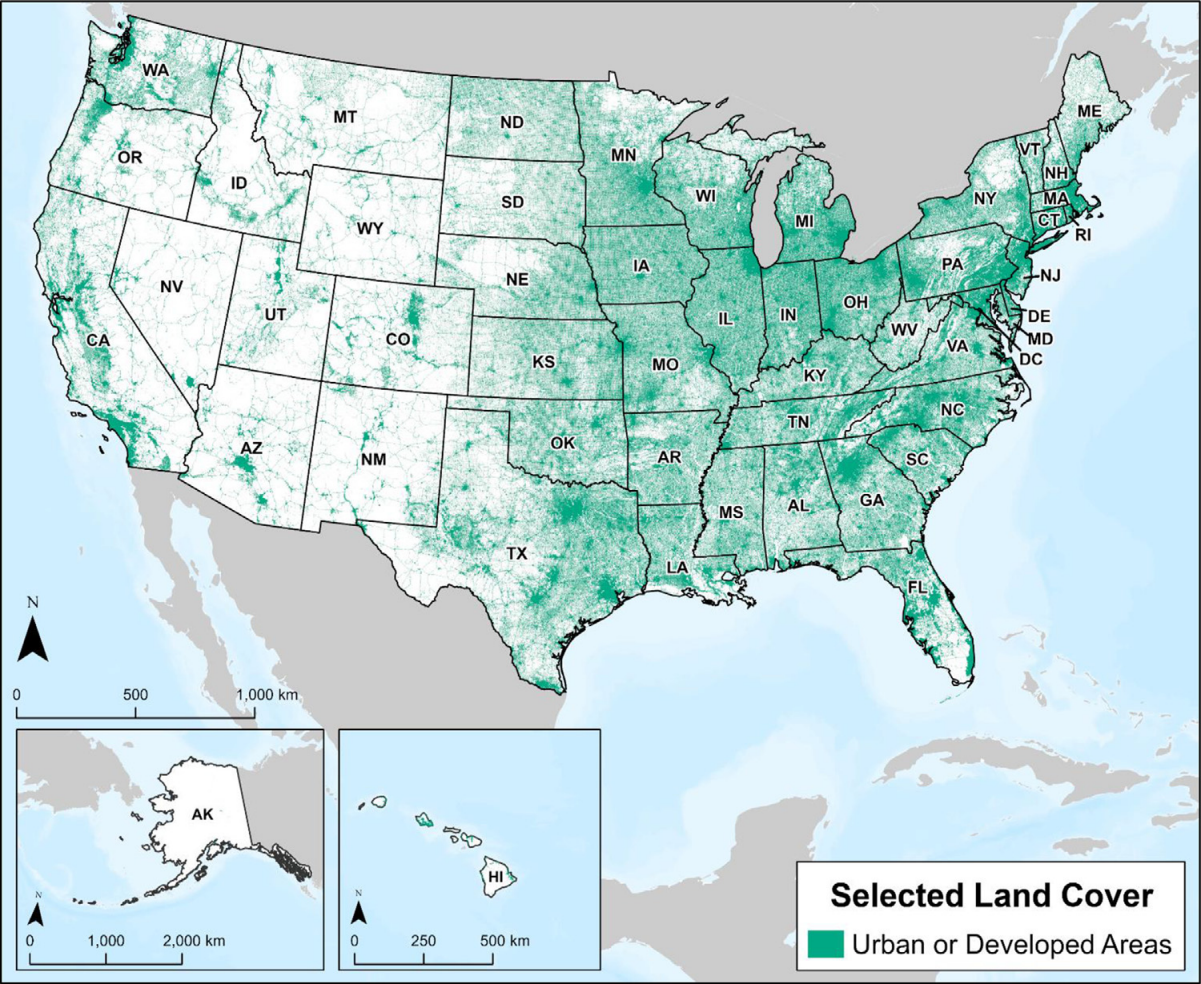
Urban or developed areas in the U.S from U.S. Census Bureau designation and selected land cover used for weighting for CO_2_ pipeline routing.

The urban and developed areas layer encompasses developed areas, cities, towns, and areas with large, high-density populations. As shown in Fig. 2, this layer is a compilation of urban boundaries from the USCB and developed areas of low, medium, or high intensity from the National Land Cover Database (NLCD) [1]. The USCB classifies urban areas by identifying census blocks with criteria laid out in 87 FR 16706 [10], including population density, housing units, and impervious surfaces. The NLCD, produced by the USGS and the Multi-Resolution Land Characteristics consortium provides land cover designations for the U.S. Federal regulations [1,3] recommend up to 304.8 m (1,000 ft) setback distance from high consequence areas (HCAs) for pipelines, thus the urban and developed areas layer includes the recommended setback distances.

#### 3.1.8 Land Cover Classified for CO_2_ Routing

**Fig. 3:**
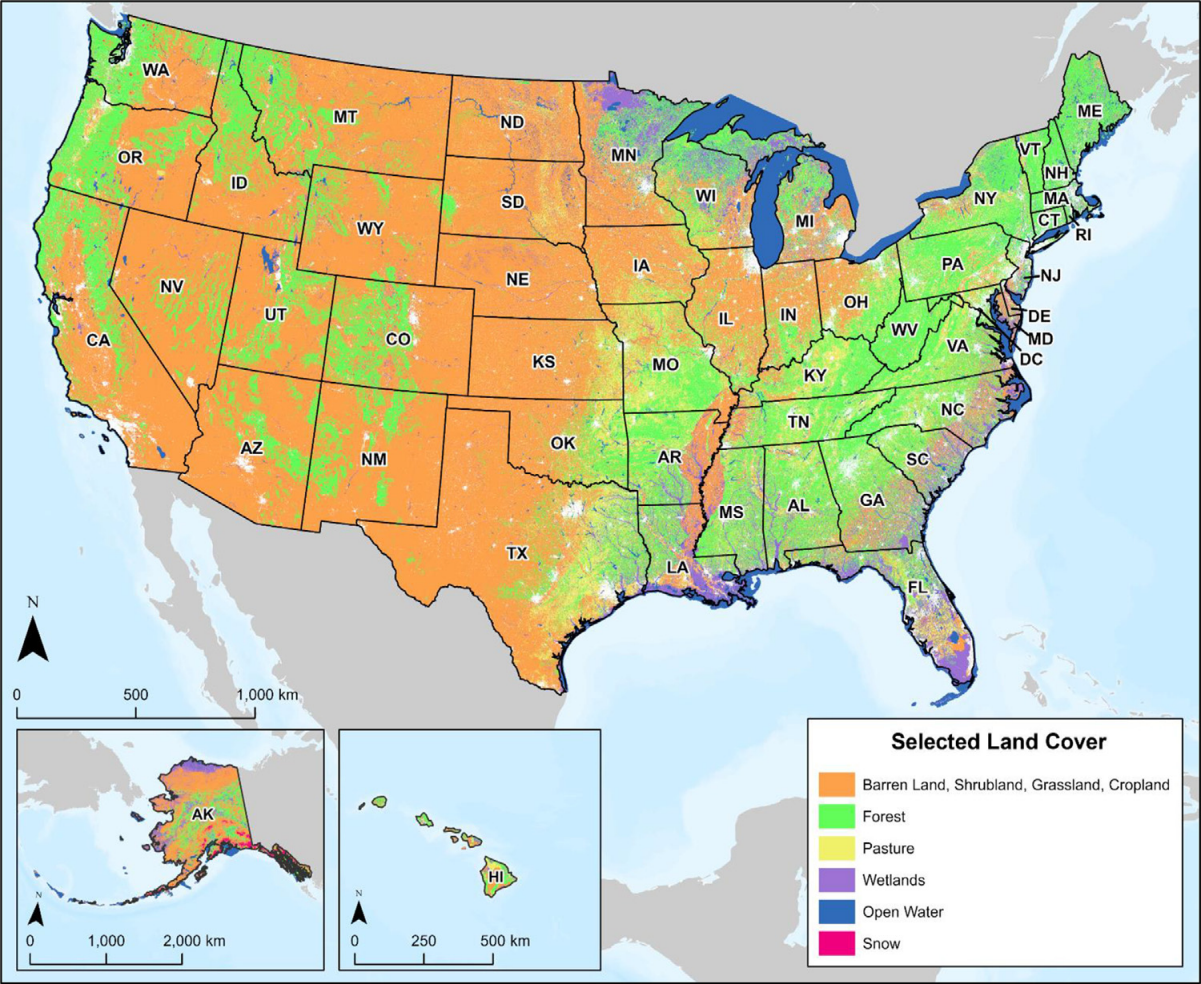
Selected land cover across the U.S. used for weighting for CO_2_ pipeline routing.

Land cover classification data were acquired from the USGS NLCD for the contiguous U.S. and Alaska, and the National Oceanic and Atmospheric Administration's National Ocean Service Office of Coastal Management for Hawaii (Fig. 3). The resulting land cover layer represents a one kilometer-squared resolution vector dataset, where each area represents a landcover designation (e.g., open water, wetland, forest). Land classifications were weighted for transport routing based on previous studies, installation cost, and best-practice recommendations for pipeline routing [1]. Land cover classified as developed has been removed from the larger land classification layer (Fig. 3) and integrated into the urban and developed areas layer (Fig. 2). The classified layer was derived utilizing the methodology of CostMAP, a cost surface multi-layer aggregation software developed by Los Alamos National Laboratory to support route optimization for energy-infrastructure modeling [11].

### 3.2 CCS by State

**Fig. 4:**
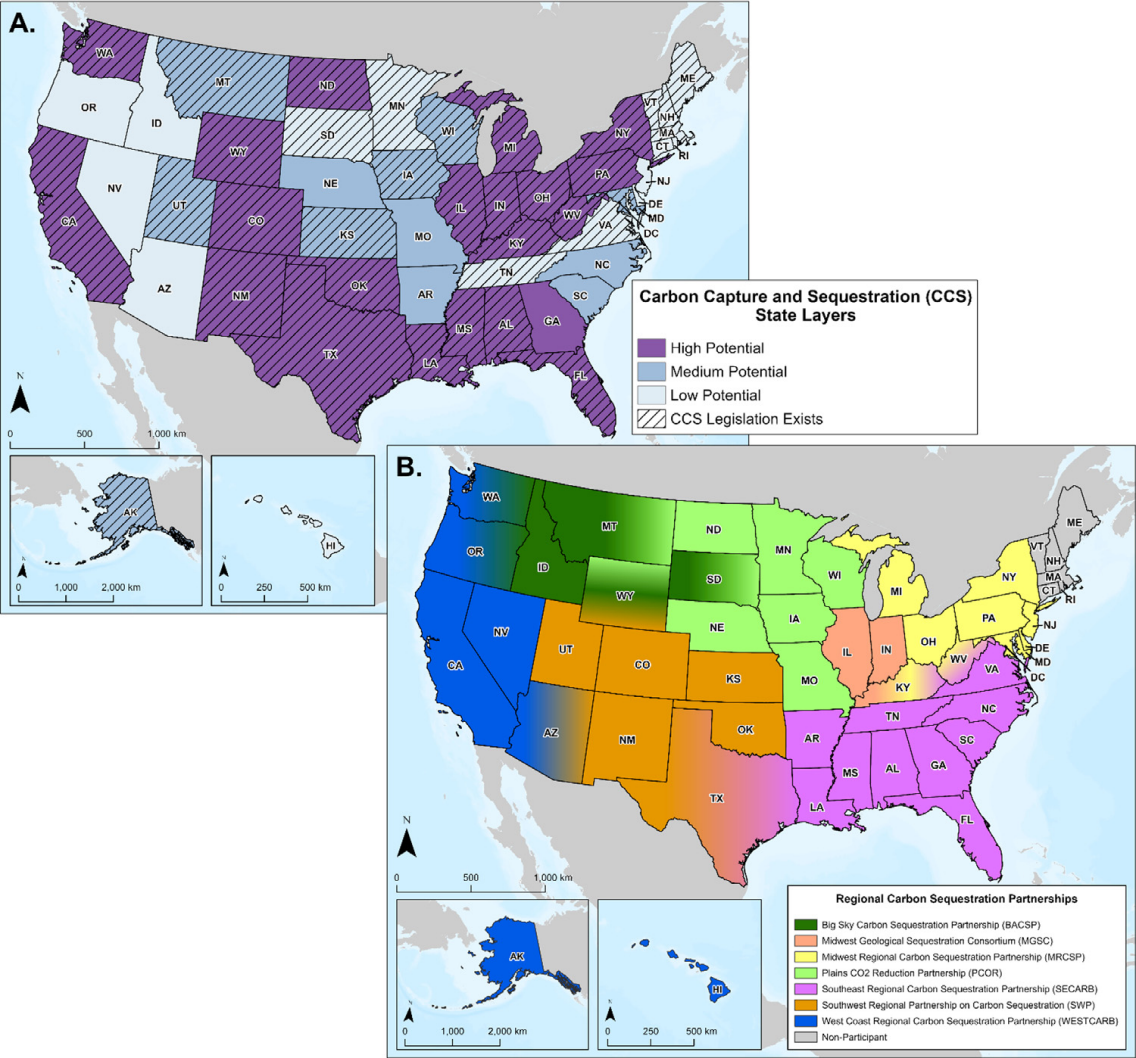
(A.) Map displaying CCS potential by state and CCS legislation availability. (B.) Map illustrating state participation in none, or one or more DOE Regional Carbon Sequestration Partnerships (RCSP).

Three layers representing CCS data and information at the state-level are provided in the database (Fig. 4). Data used to create these layers were derived from existing U.S. Department of Energy (DOE) research and analysis of CCS development potential, regional carbon storage initiative participation, and existing or pending legislation.

#### 3.2.1 CO_2_ Atlas CCS Potential by State

The National Carbon Sequestration Atlas (NATCARB) from DOE's National Energy Technology Laboratory (NETL) provides information on stationary source emissions and prospective geologic storage resource estimates by state [12]. A spatial layer representing CCS potential per state was created based on annual CO_2_ emissions, number of emission sources, and median prospective storage resource storage estimates (Fig. 4A). Each of the aforementioned factors were ranked and summarized with the top third of all states considered to have high CCS potential, the next third ranked as medium CCS potential, and the bottom third ranked as low CCS potential [1].

#### 3.2.2 CO_2_ Infrastructure Legislation Availability by State

The Legislation Availability by State layer represents a static snapshot of whether a state has existing CCS-related legislation, as of March 2023 (Fig. 4A). Legislation might include regulations of injection sites or pipelines, directives to create CCS taskforces or programs, or tax incentives. A detailed list of the legislation available by state is available in the metadata excel sheet alongside the CCS Pipeline Route Planning Database [1].

#### 3.2.3 CO2 Taskforce Involvement by State

The CO_2_ Taskforce Involvement by State layer denotes whether a state was a participant in DOE's Regional Carbon Sequestration Partnership (RCSP) initiative [13] (Fig. 4B). The RCSP network was developed to support implementing suitable carbon storage technology, infrastructure, and regulations across the U.S. and parts of Canada.

### 3.3 Environmental, Energy, and Social Justice

A key aspect of infrastructure planning is community sentiment, as public concern has inhibited CCS-related development [14]. Accordingly, EJSJ considerations during planning are pertinent for project success [15]. Layers representing various EJSJ variables were integrated to support the inclusion of socially responsible and environmentally prudent considerations when planning potential CO_2_ transport routes. These layers represent areas that have energy, social, economic, or environmental concerns including disadvantaged communities, areas of increased social or environmental vulnerability, and areas with higher likelihoods of natural disasters. Each layer was collected from authoritative sources including the Center for Disease Control (CDC), the Environmental Protection Agency (EPA), and the Federal Emergency Management Agency (FEMA) [1].

#### 3.3.1 National Risk Index

**Fig. 5:**
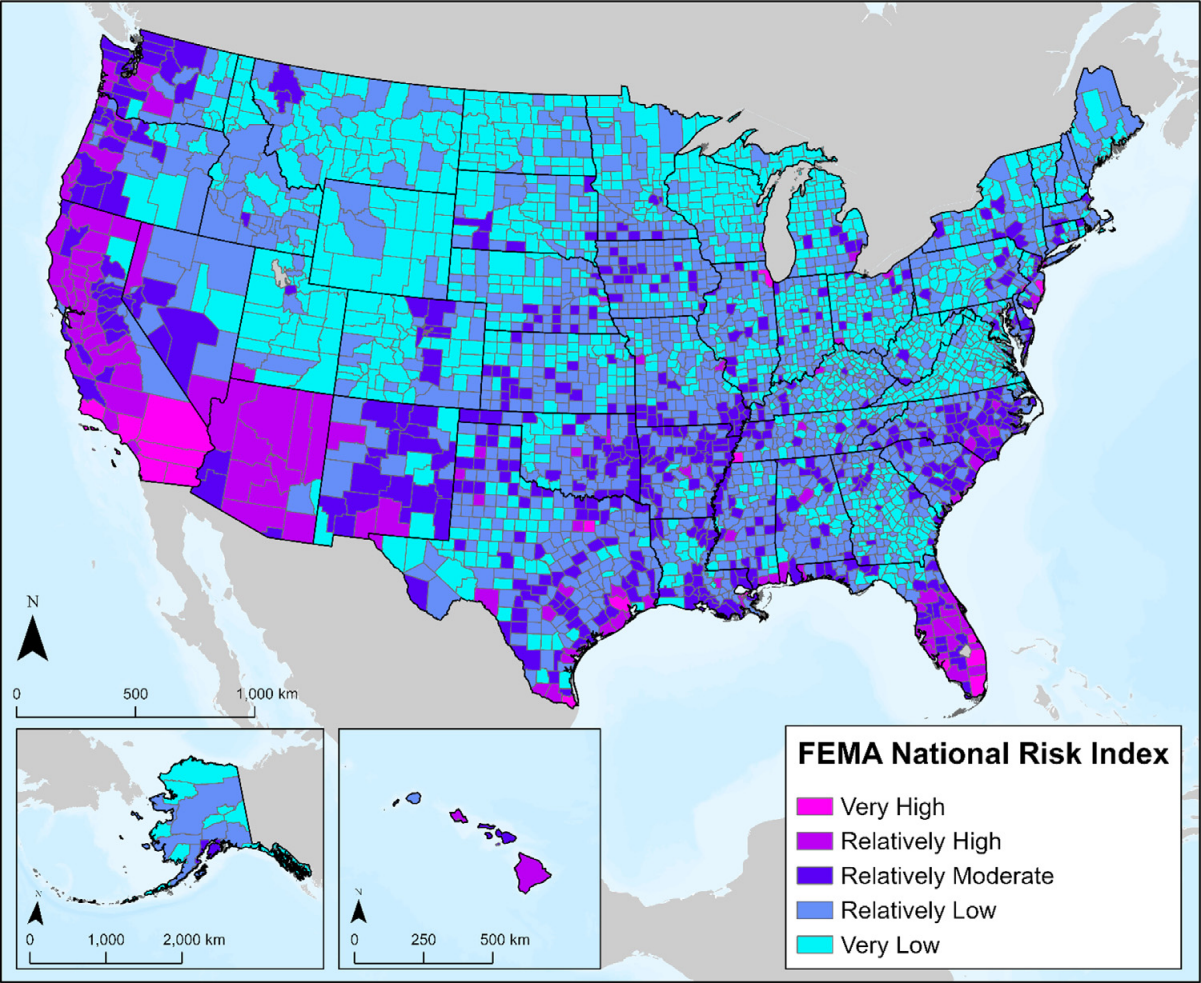
Map representing FEMA's National Risk Index ranking by county.

The National Risk Index (NRI) dataset is hosted by FEMA for U.S. communities and emergency managers to better understand risks associated with 18 distinct natural hazards [16]. Acquired at the county-level, this dataset provides a baseline risk measurement for natural hazards and social vulnerability. As illustrated in Fig. 5, the overall risk index equation represents the impact potential from natural hazards and is comprised of two components, average annual expected economic loss from natural hazards, and a scaling factor for community risk, which incorporates a measure of social vulnerability and community resilience. As such, the inclusion of this dataset acts as a measure for potential natural hazard impacts on CO_2_ transport routing infrastructure, as well as community-based vulnerability and resilience.

#### 3.3.2 Environmental Justice Screen

**Fig. 6:**
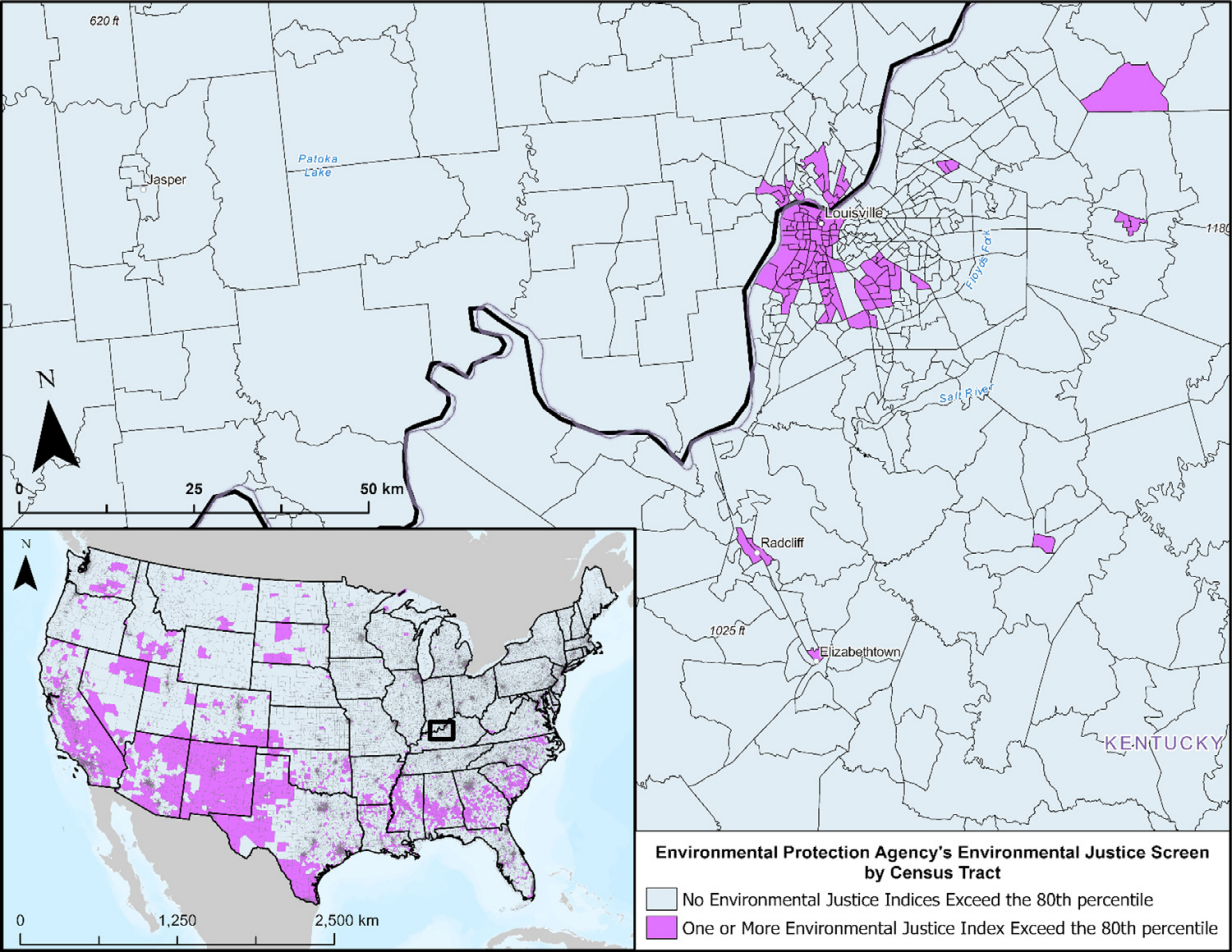
Map representing the EPA's Environmental Justice Screen (EJScreen) by census tract, highlighting areas where one or more of the EJScreen indices falls within the 80th percentile when compared to all other census tracts. This figure includes a zoom in of Louisville, Kentucky, and the surrounding area. Data are also available for Alaska and Hawaii.

The EPA developed the Environmental Justice Screen (EJScreen) tool to address and quantify environmental justice (EJ) across the country [1]. Provided at the census tract-level, EJScreen data were developed using a methodology to consolidate environmental and demographic indicators into EJ indices, thereby providing a holistic understanding of the interplay between environmental factors and socioeconomic dynamics. EJScreen encompasses 13 environmental indicators (e.g., wastewater discharge, lead paint, traffic proximity) and seven socioeconomic indicators (e.g., people of color, low-income), which collectively contribute to the calculation of 13 EJ indices (e.g., traffic indicator, people of color populations) and 13 supplemental indices (e.g., percent of low income, low life expectancy). As shown in Fig. 6, EJScreen data were integrated into the CCS Pipeline Route Planning Database to account for key EJSJ considerations.

#### 3.3.3 Environmental Justice Index

**Fig. 7:**
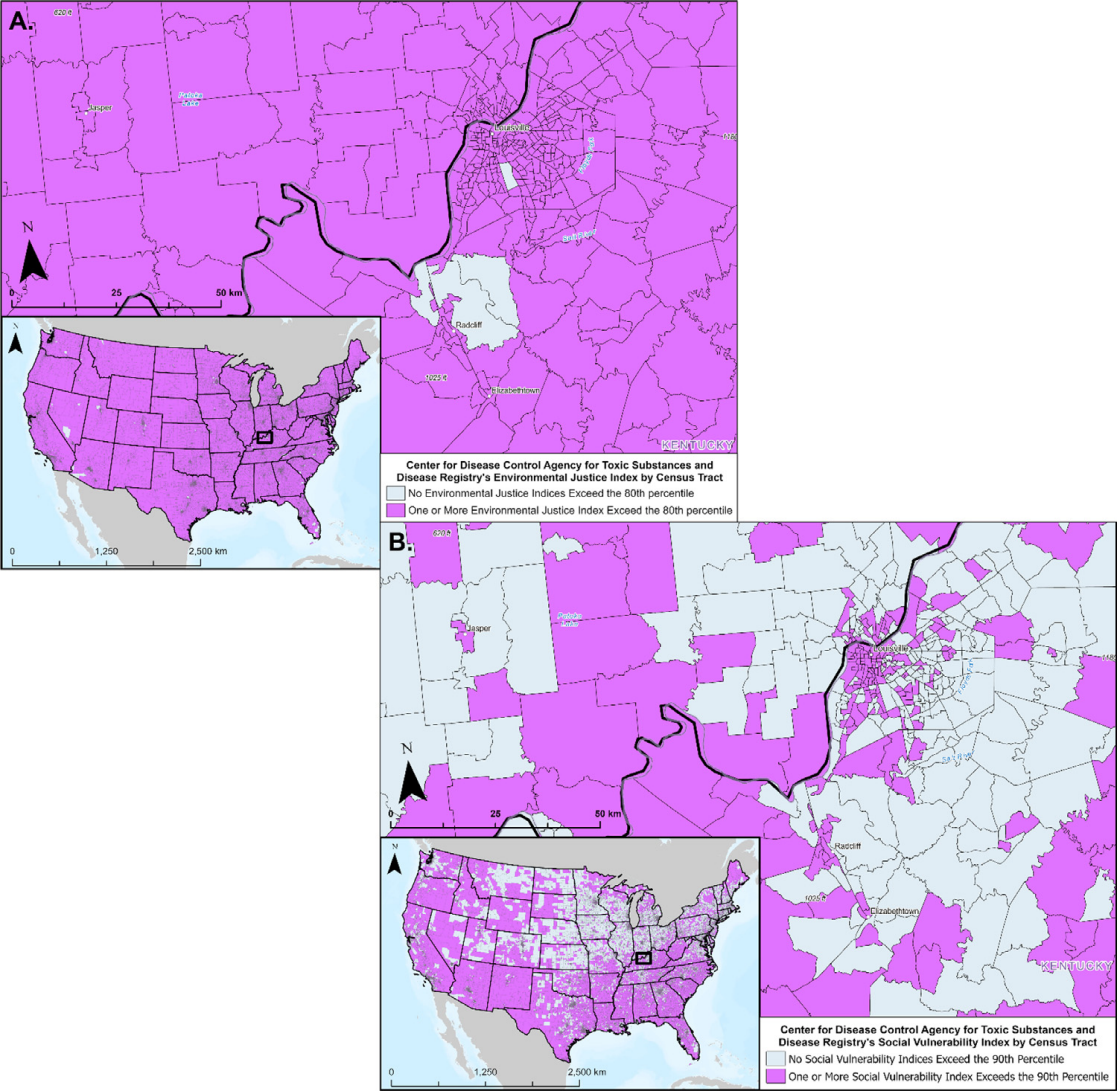
Maps A and B show examples of the CDC's Agency for Toxic Substances and Disease Registry's (ATSDR) Environmental Justice Index (EJI) and Social Vulnerability Index (SVI) by census tract, with a zoomed in view of Louisville Kentucky and the surrounding area. Data are also available for Alaska and Hawaii. (A.) Map displaying EJI data, highlighting areas where one or more of the EJI indices fall within the 80th percentile when compared to all other census tracts. (B.) Map displaying SVI data, delineating areas where one or more of the SVI indices fall within the 90th percentile when compared to all other census tracts.

The CDC Agency for Toxic Substances and Disease Registry's (ATSDR) developed the Environmental Justice Index (EJI) using data from the CDC, USCB, EPA, and the U.S. Mine Safety and Health Administration to assess and rank the cumulative impacts of environmental injustice on public health [1]. Accounting for 36 environmental, social, and health factors (e.g., household characteristics, socioeconomic status, potentially hazardous and toxic sites, transportation infrastructure, estimated prevalence of asthma) the resulting EJI provides a comprehensive overview of patterns of environmental injustice across the U.S. As displayed in Fig. 7A, the EJI dataset was incorporated into the CCS Pipeline Route Planning Database to account for EJ considerations for CO_2_ transport-route planning.

#### 3.3.4 Social Vulnerability Index

The CDC ATSDR's Social Vulnerability Index (SVI) is a comprehensive dataset that measures community likelihood for requiring assistance during public health emergencies [1]. A measure of relative vulnerability, SVI ranks census tracts based on 16 social factors (e.g., unemployment, ethnic minority status, disability), which are subsequently categorized into four interconnected themes, socio-economic status, household characteristics, racial and ethnic minority status, and housing type and transportation. Built to inform effective resource prioritization and allocation during public health emergencies, the CDC SVI data accounts for social justice considerations when planning CO_2_ transport routes (Fig. 7B).

#### 3.3.5 Disadvantaged Communities

**Fig. 8:**
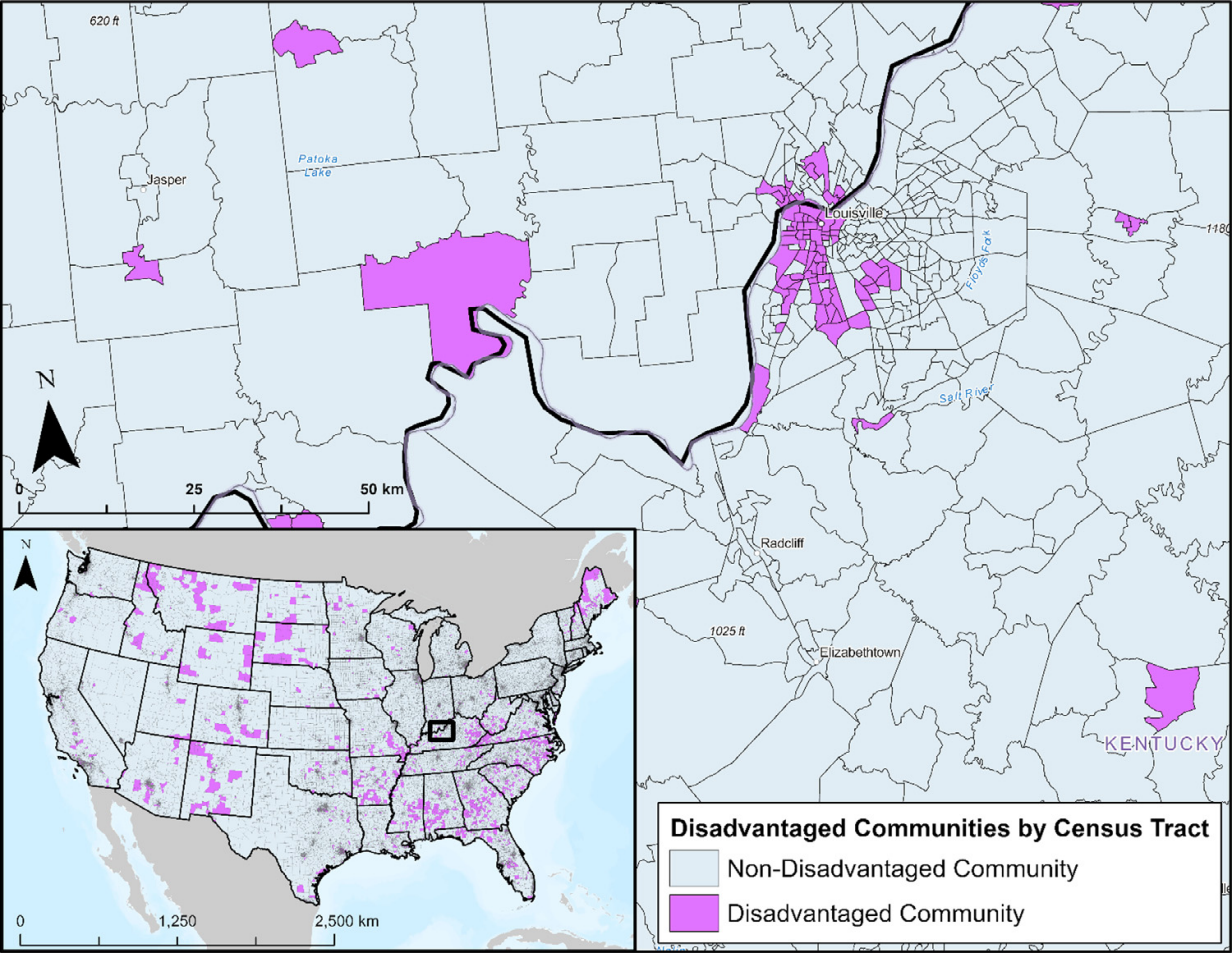
Map representing disadvantaged communities. Disadvantaged communities are defined by DOE as census tracts that are in the 80^th^ percentile for the cumulative burden indicators and have at least 30% of households classified as low-income. This figure includes a zoom in of Louisville, Kentucky, and the surrounding area. Data are also available for Alaska and Hawaii.

The Justice40 Initiative, which was created following the 2021 Executive Order entitled “Tackling the Climate Crisis at Home and Abroad”, was designed to ensure that disadvantaged communities receive 40 % of funding from certain federal investments [17]. As a result, DOE developed a geospatial tool to categorize disadvantaged communities based on cumulative burden of 36 indicators reflecting energy burden, environmental hazards, socio-economic vulnerabilities, and fossil dependence. A layer representing these disadvantaged communities, provided at the census tract level as shown in Fig. 8, are included in the database for planning consideration.

### 3.4 Hydrology

Hydrology layers represent both underground and surface water resources. These are included because of potential risks of cross-contamination by CO_2_, making it a critical consideration when planning carbon transport routes. Hydrology data were collected from three USGS resources.

#### 3.4.1 National Hydrology Dataset (Wells, Waterbodies, Areas, Flowlines)

**Fig. 9:**
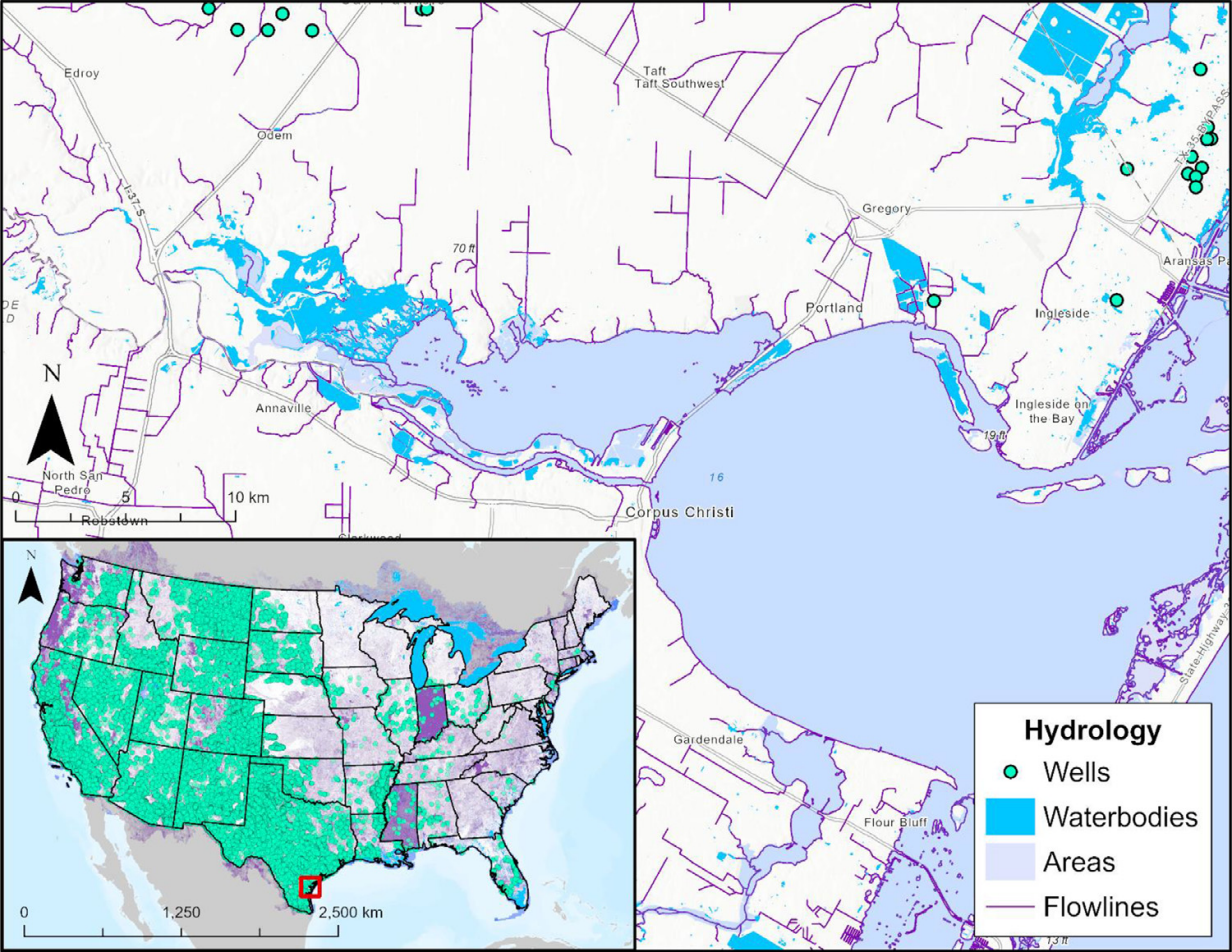
Map representing the USGS National Hydrology Database (NHD) datasets, which are included in the CCS Pipeline Route Planning Database. This figure includes a zoom in of Corpus Christi, Texas, and the surrounding area.

As shown in Fig. 9, four data layers were gathered and revised from the USGS National Hydrology Dataset (NHD) [1], representing information on water-related resources across the U.S. These include water wells with a 100-foot setback distance, based on petroleum product recommendations from the CDC, Colorado, and Wisconsin [1]. Flowlines were also added as representations of streams, rivers, artificial paths, canals, ditches, pipelines, and coastlines. Lastly, waterbodies and areas representing lakes, ponds, and supplemental water features were added to the database. These layers are critical for route planning, as wetland and waterbody construction and mitigation procedures from the Federal Energy Regulatory Commission (FERC) state that it is best to avoid water crossings whenever possible [1].

#### 3.4.2 Aquifers

**Fig. 10:**
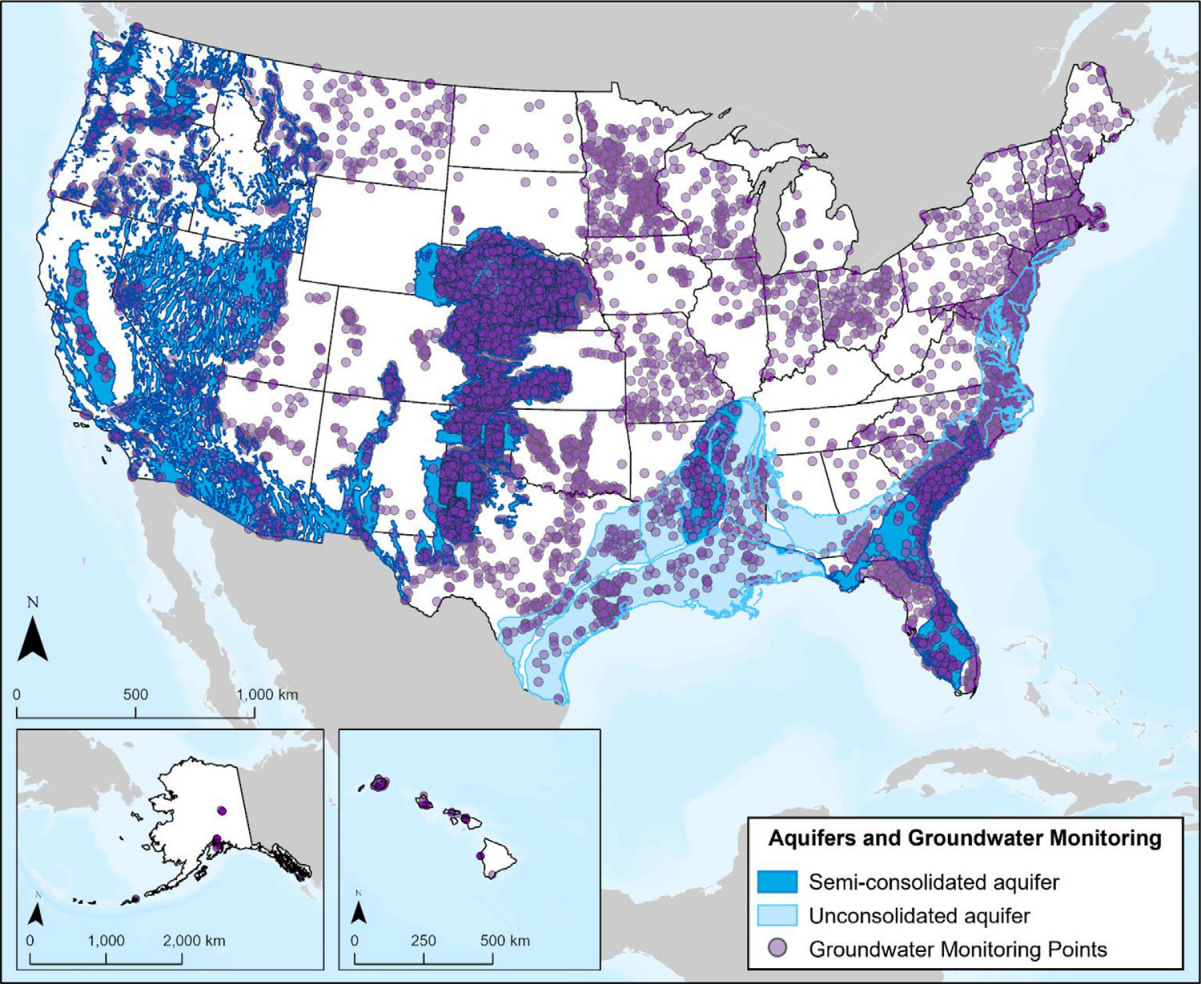
Map displaying semi-consolidated and unconsolidated aquifers across the contiguous U.S., as well as groundwater monitoring site locations across the U.S., including Alaska and Hawaii.

The Ground Water Atlas of the U.S., prepared by the USGS, includes spatial representations of shallow (i.e., near-surface) principal aquifers in the contiguous U.S. [1]. The aquifers layer represents an interpretation of the surface or near-surface area of the uppermost principal aquifer; therefore, the actual aquifer might be larger than those represented in Fig. 10. From the original resource, semi-consolidated and unconsolidated aquifers were selected for inclusion in the database due to the potential for contamination from pipeline leaks or failures. For instance, in South Dakota, there are stricter requirements for clean-up and monitoring for oil and gas spills that occur in unconfined aquifer areas, than those that are confined [1]. As CO_2_ regulations have not yet been established in relation to aquifers, petroleum pipeline recommendations were consulted.

#### 3.4.3 Groundwater Monitoring Locations

Groundwater monitoring locations were acquired from USGS and represent monitoring wells across the U.S. [1] (Fig. 10). This layer was processed to represent groundwater monitoring well locations, including information on well ownership (USGS or state), site number, site name, location represented as latitude and longitude, well altitude, well depth, type of aquifer, and a link to the USGS Ground Water Monitoring web application for additional information.

### 3.5 Infrastructure

This category contains layers representing transportation, energy, and public infrastructure. Infrastructure layers have specific regulations detailed in 49 CFR § 195 concerning the construction of hazardous liquid pipelines [3], which might include depth, setback, or cover requirements.

#### 3.5.1 Roads

**Fig. 11:**
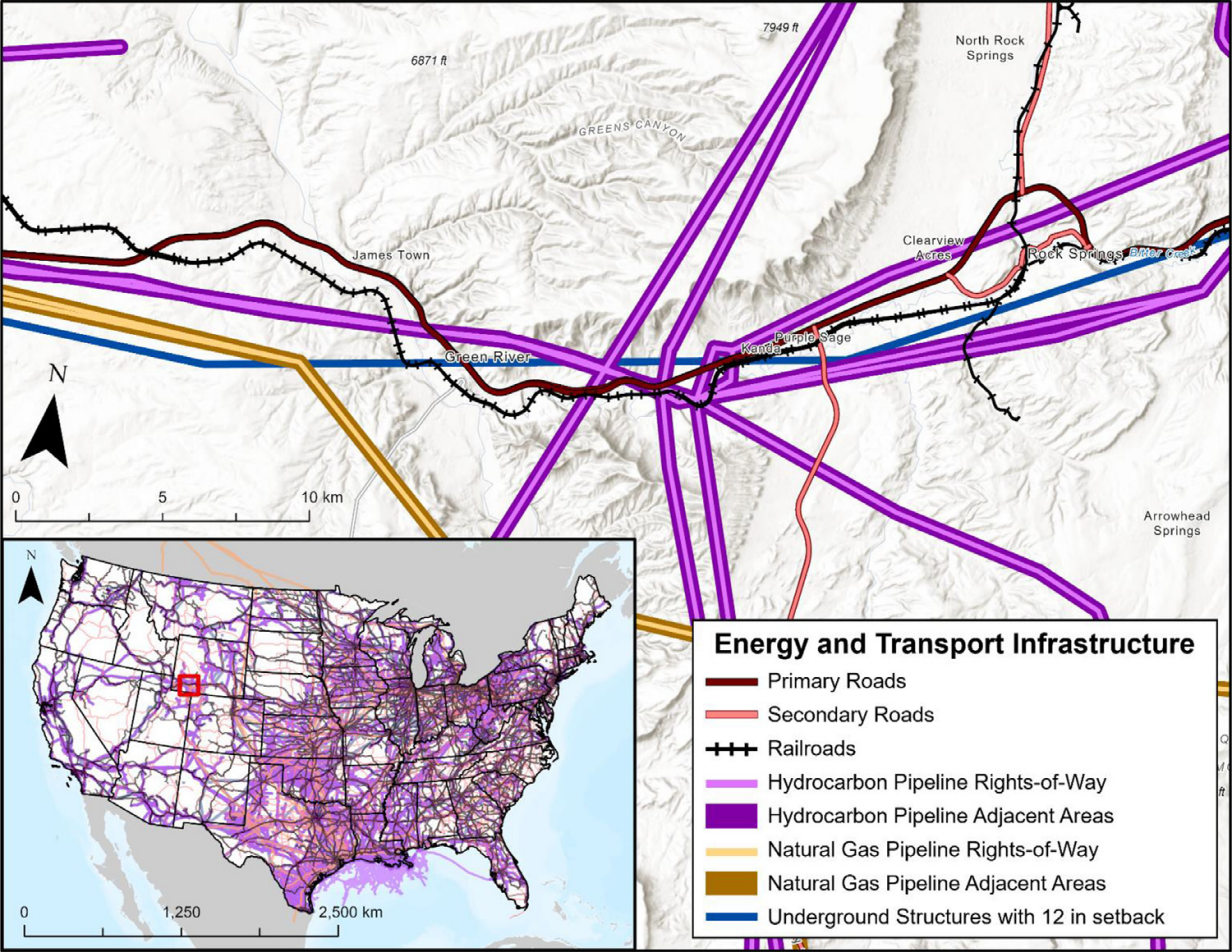
Map displaying energy and transport infrastructure including roads, railroads, underground structures, and pipeline ROWs or adjacent areas. This figure includes a zoom in of Rock Springs, Wyoming, which highlights the intersection of energy and public transport infrastructure, natural gas, and hydrocarbon pipelines alongside underground structures.

The database includes three road infrastructure layers spanning the U.S. [1]. Primary and secondary roads, representing interstates and highways, were acquired from the U.S. Department of Homeland Security's (DHS) Homeland Infrastructure Foundation-Level Data online platform (HIFLD) (Fig. 11). In addition, a local roads layer was collected from the USCB [1]. Following previous study findings, road layers include setback distances of 30.48 m (100 ft) for all road types [2]. Best pipeline construction practices note that crossing of roads is recommended against, but when it cannot be avoided, additional construction requirements are enforced [1]. Relevant guidelines for intersections of road and pipeline infrastructure include 49 CFR § 195.256, which notes that pipelines must be installed in such a way that it can withstand the dynamic forces of the anticipated traffic load, and 49 CFR § 195.248(a) which includes cover over buried pipeline requirements, including areas under roadbeds and drainage ditches [3].

#### 3.5.2 Railroads

The railroads layer represents a combination of data from the USCB and acquired through HIFLD [1] (Fig. 11). Based on past research, the railroad layer includes setback distances of 30.48 m (100 ft) [2]. Like roads, best pipeline construction practices note that crossing of railway infrastructure is recommended against, but when it cannot be avoided, additional construction requirements are enforced [1]. These requirements include 49 CFR § 195.256, which provides guidance that pipelines at railroad crossings must be installed in such a way that it can withstand the dynamic forces of the anticipated traffic load for that railroad [3]. In addition, 49 CFR § 195.248(a) provides cover over buried pipeline requirements, including drainage ditches at public railroad locations [3].

#### 3.5.3 Hydrocarbon and Natural Gas Pipeline Right-of-Ways & Adjacent Areas

The database contains four pipeline layers representing ROWs for hydrocarbon and natural gas pipelines, as well as areas surrounding ROWs (Fig. 11). Areas surrounding ROWs were included as pipeline placement within those existing corridors might offer a cost-effective option as they should already meet fossil energy pipeline regulations, though might not be appropriate for CO_2_ pipeline placement. The hydrocarbon pipeline layer and surrounding areas started as a composite of hydrocarbon gas liquid pipeline data from the U.S. Energy Information Administration (EIA) and HIFLD [1]. The natural gas pipeline layer and surrounding areas layer began as integrated natural gas pipeline data from both the Oak Ridge National Laboratory (ORNL) and HIFLD [1]. Setback distances of 15.24 m (50 ft) were applied to the resulting pipeline locations to represent an estimated distance of associated ROWs, with 7.62 m (25 ft) on either side of the pipeline [1]. For the areas surrounding pipelines, ROWs were spatially buffered by an additional 91.44 m (300 ft), then the areas representing ROWs were spatially removed from the now 198.12 m (650 ft) wide areas, resulting in areas surrounding, but not including ROWs [2].

#### 3.5.4 Underground Structures

The underground structures layer represents coal mines, underground natural gas storage, petroleum product pipelines, and natural gas pipelines. The EIA provides publicly available layers online, which were acquired and compiled to produce one representative layer [1]. The underground structures layer represents federal regulations laid out in 49 CFR § 195 state that underground pipelines must be given a 305 mm (12 inch) setback distance from any other underground structure in most cases [3] (Fig. 11).

#### 3.5.5 Public & Private Infrastructure

**Fig. 12:**
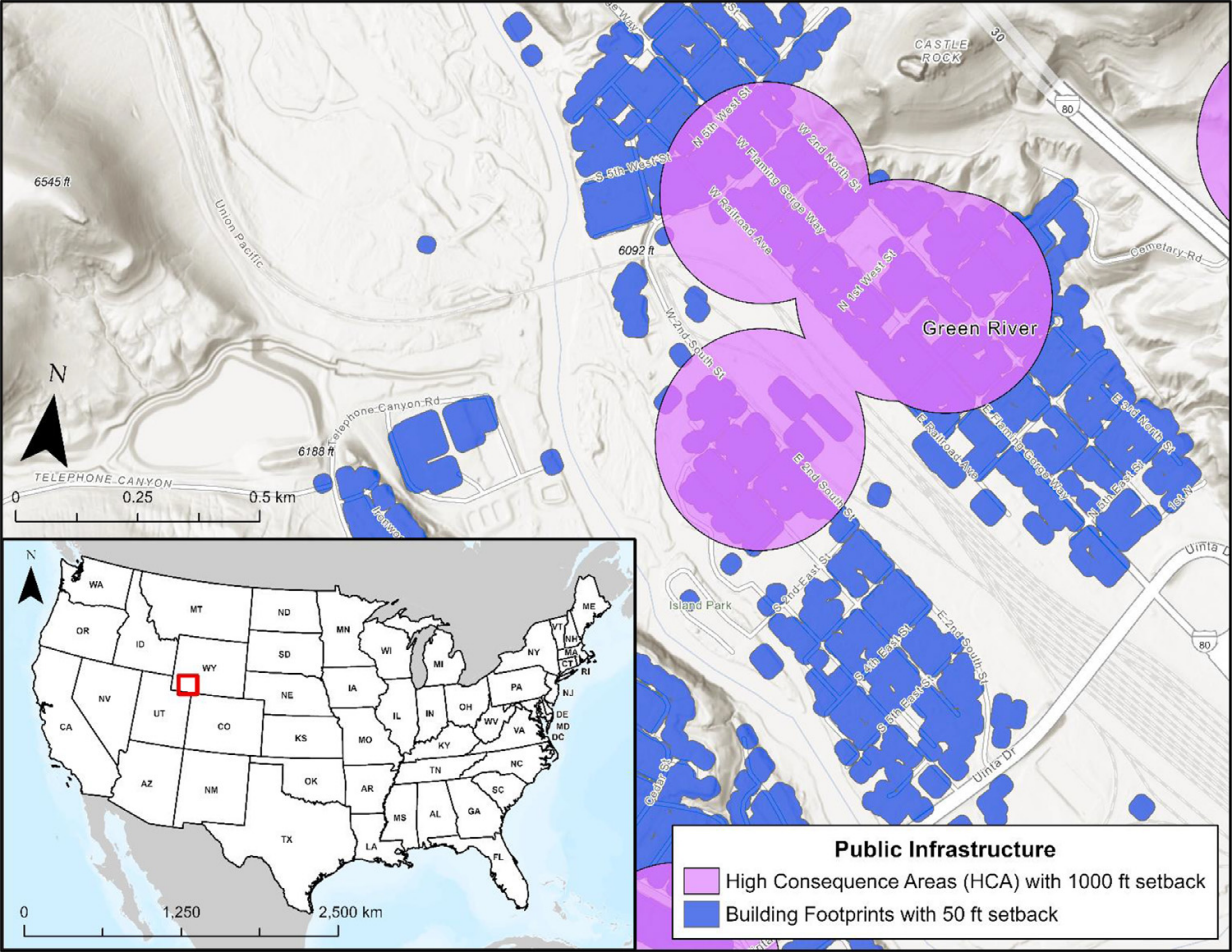
Map of Green River, Wyoming illustrating public infrastructure layers including HCAs and building footprints, which are available across the U.S., including Hawaii, Alaska, and U.S. territories.

Public and private building infrastructure, including HCAs, are represented in the buildings and public infrastructure layers within the CCS Pipeline Route Planning Database (Fig. 12). These layers represent areas of buildings and associated setback distances of 15.24 – 304.8 m (50 – 1,000 ft), based on federal regulations for petroleum and CO_2_ pipelines and ROWs [3,18]. Nationwide building footprint data representing critical infrastructure and residential buildings greater than 137.16 m^2^ (450 ft^2^) were acquired from a collaborative effort among FEMA, DHS Science and Technology Directorate, and ORNL [1]. Layers included in the database represent federal regulations on petroleum pipelines, which note that pipelines cannot be located within 15.24 m (50 ft) of private dwellings, industrial buildings, or any place where the public might work, congregate, or assemble without additional construction requirements [3]. Public structures, including hospitals, schools, and fire and police departments, were acquired through the USGS and represent HCAs with setbacks of 304.8 m (1,000 ft). The applied setback is based on a 2022 gas transmission pipeline regulation, which requires 300-, 660-, or 1,000-feet setbacks for HCAs depending on pipe diameter and operating pressure [1,18]. The most conservative distance was applied.

### 3.6 Natural Hazards

Natural hazards encompass layers representing potential geohazard or extreme weather risks, including slope, landslide susceptibility, soil considerations, FEMA's NRI natural hazards, and floodplains. Each of these layers have specific regulations detailed in 49 CFR § 195 concerning the construction and inspection of hazardous liquid pipelines [3]. Regulations include corrosion control, cover requirements, or guidance on pipeline inspection procedures after extreme weather and natural disasters.

#### 3.6.1 Landslide Susceptibility

**Fig. 13:**
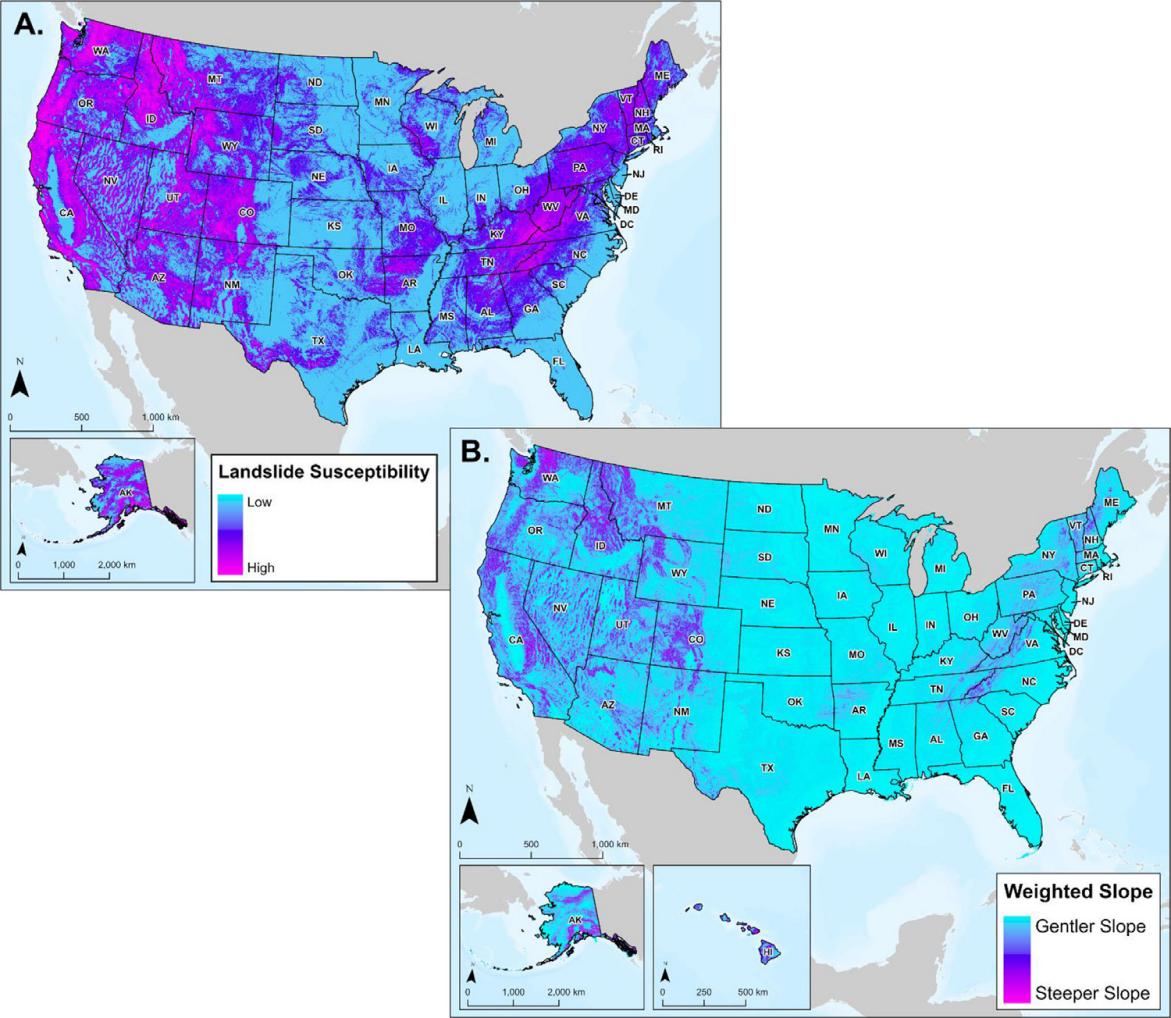
(A.) Landslide susceptibility ranked from low to high. Landslide susceptibility data were not available for Hawaii. (B.) Weighted slope from low (gentler) to high (steeper) spanning the U.S.

Understanding landslide susceptibility is critical for mitigating potential impacts to current and prospective infrastructure. As such, the global landslide susceptibility dataset developed by the National Aeronautics and Space Administration (NASA) was integrated into the routing database [1]. The method NASA applied to classify landslide susceptibility incorporated slope, distance to fault zones, geologic classifications, presence of roads, and forest loss, which resulted in five rankings: very low, low, medium, high, and very high (displayed as continuous color scale in Fig. 13A).

#### 3.6.2 Weighted Slope

Slope, or change in elevation, is a major concern for pipeline route planning as areas with steep slopes are typically at increased risk of natural hazards such as landslides or debris flows, which can damage or destroy in situ infrastructure [16]. Operators are required by 49 CFR § 192.31(a) to implement practical measures to safeguard every transmission line or main against potential damage caused by washouts, floods, unstable soils, landslides, or any other hazards that could lead to the pipeline moving or experiencing excessive stress [18]. The weighted slope layer in the CCS Pipeline Route Planning Database is a vector surface derived from the USGS 3D Elevation Program's digital elevation model [1]. As shown in Fig. 13B, the weighted slope layer represents areas of slope unsuitable for pipeline placement (greater than or equal to 30 % grades), and areas with flatter grades providing potentially safer routing opportunities [1].

#### 3.6.3 Soil Frost Action and Corrosion Potential

**Fig. 14:**
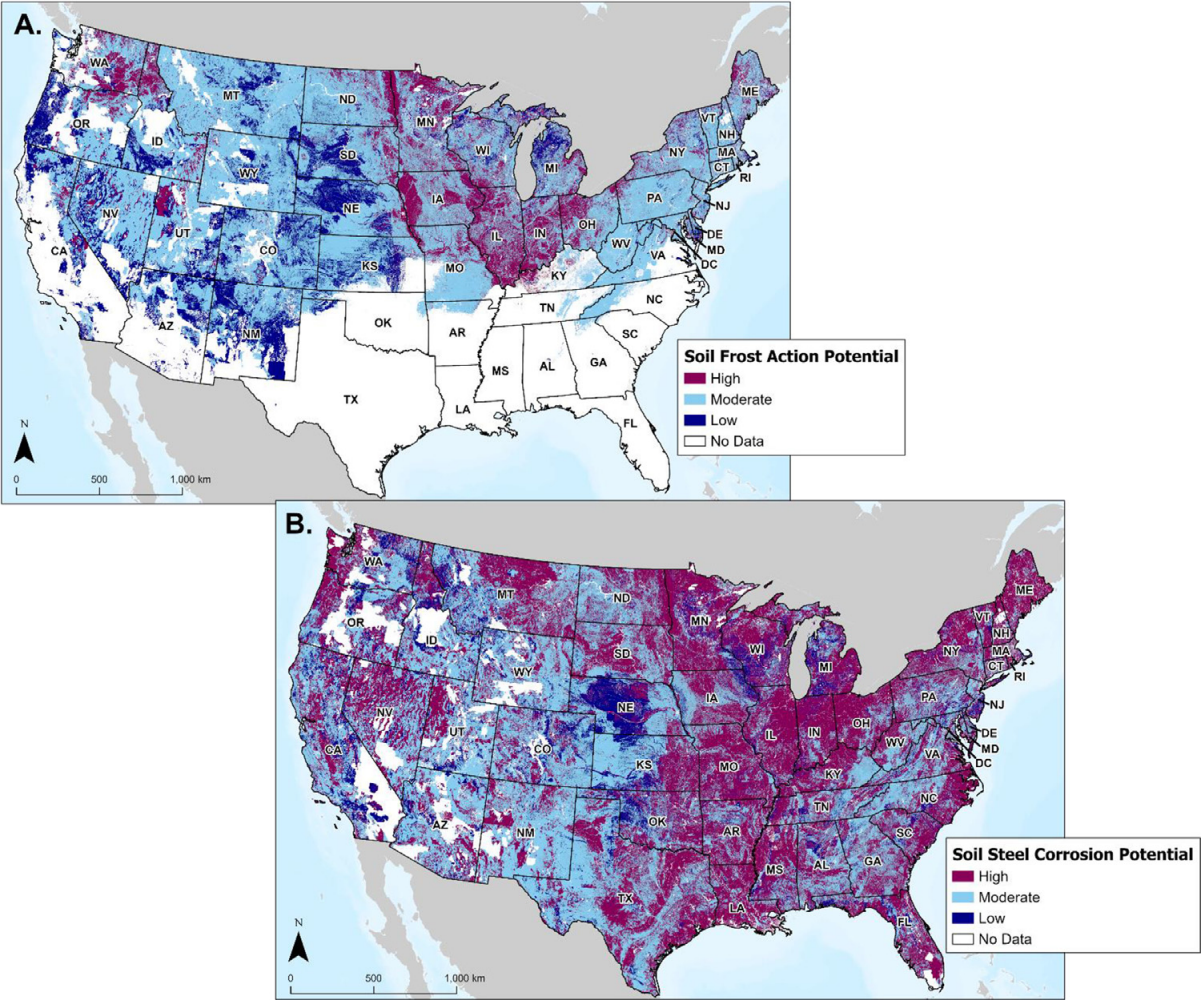
(A.) Map displaying soil frost action potential for the contiguous U.S. (B.) Map displaying soil steel corrosion potential for the contiguous U.S.

Pipeline infrastructure may be susceptible to damage or failure due to key soil conditions, therefore locating potentially hazardous soil conditions are critical for deploying preventative maintenance and monitoring practices. Soil attribute data from the Soil Survey Geographic Database (SSURGO) made available by the U.S. Department of Agriculture-National Resources Conservation Service were aggregated into soil map units for the U.S. key soil attributes that represent potential hazards are frost action (i.e., frost heaving) and (uncoated) steel corrosion potential [1]. Frost action has the potential to physically damage infrastructure when soil freezes, while corrosion damage may chemically interact with and thin or penetrate steel pipeline walls. As shown in Fig. 14, layers representing both hazards were included in the database with spatial classifications of low, moderate, and high potential. For corrosion, these categories were ranked based on soil drainage class, acidity, and electrical conductivity values, while frost action is based on soil moisture regime and particle size classes [1].

#### 3.6.4 Natural Hazard National Risk Indices

As described previously, the FEMA NRI provides critical natural hazard information integrated with social and environmental factors. Specific natural hazard risk indices were selected to be incorporated into the CCS Pipeline Route Planning Database, these include earthquake risk, riverine and coastal flood risk, wildfire risk, and landslide risk. All county index scores range from 0 to 100, based on the given value's percentile ranking in the national distribution.

#### 3.6.5 Floodplains

**Fig. 15:**
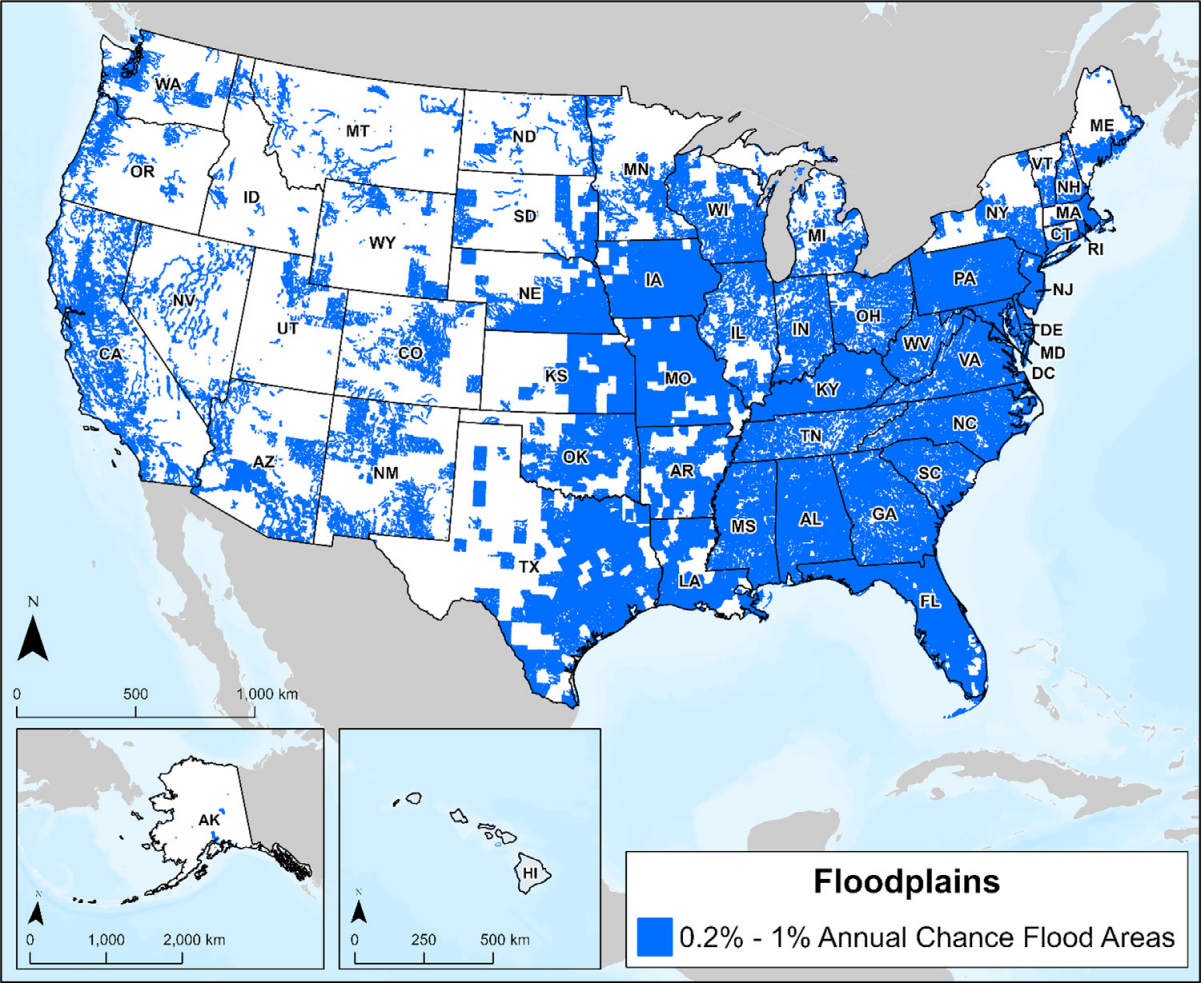
Floodplain map of the U.S. representing areas with a 0.2 % – 1 % annual flood chance.

Floods have historically damaged and destroyed pipelines [19], therefore national floodplain data were integrated to account for potential flood susceptibility to support safe and sustainable route planning. The National Flood Hazard Layer (NFHL), maintained by FEMA, is a geospatial database built to support the National Flood Insurance Program (NFIP) by providing up-to-date and reliable flood hazard information [1]. The NFHL was acquired to represent potential flood hazards, including riverine, coastal, and flash flooding. The floodplains layer in the CCS Pipeline Route Planning Database represents a subset of the NFHL data where annual chance of floods ranges from 0.2% to 1% (Fig. 15).

## 4 Experimental Design, Materials, and Methods

The CCS Pipeline Route Planning Database was designed to serve as a comprehensive geospatial resource to support safe CO_2_ pipeline route planning, and act as a key input into an upcoming CO_2_ transport-route planning tool, which will identify optimal routes from source to sink utilizing machine learning and spatial analytics. Given this scope, data needs were identified through a comprehensive literature review, which included state and federal regulations and legislation, as well as reports identifying common pipeline stressors, EJSJ considerations, and complimentary energy routing efforts. Data needs were cataloged and prioritized, along with preliminary weighting schemas based on literature review findings.

Next, manual data searches were conducted to acquire publicly available data representing the aforementioned needs from credible online sources. A key objective of this process was to identify and acquire the most current, complete, and accurate datasets possible. In some cases, datasets from multiple sources were combined to provide better coverage and additional insights (i.e., urban or developed areas layer). Moreover, some layers were built in-house to map key CO_2_ route planning considerations (i.e., CCS by State Legislation). Following the acquisition or creation of data, all layers were checked for quality and completeness. In parallel with data acquisition, metadata on each resource were cataloged, including source citation, link to the original resource, and data last updated and acquired [1]. Additional fields were added to the catalog linking key literature, regulations, and legislation per relevant dataset.

Each dataset was processed and had preliminary weights applied prior to the inclusion in the CCS Pipeline Route Planning Database. Given the disparate nature of the original datasets, processing needs varied by resource. Processing steps included querying datasets spatially or by attribute (i.e., land cover type), adding setback distances (i.e., spatially buffers) based on specific regulation, legislation, or best-practice guidelines, or spatially merging datasets (i.e., urban areas and developed areas). In addition, layers which were acquired as raster or non-spatial formats (e.g., comma separated value file) were converted to vector formats (i.e., point, line, polygon). Larger datasets (i.e., those containing more than one million individual features) were converted to multi-part geometries and were simplified. An example of simplification includes spatial geometries or topology of multiple features in a specific dataset overlapped at any one location, those features were spatially dissolved into a single feature. When dissolving processes were appropriate, attributes (i.e., fields) within datasets that were directly related to the weighting schema were retained for future weight adjustments. All layers were transformed into WGS 1984 Web Mercator (Auxiliary Sphere), then categorized into common themes (Table 1). Processing was completed using geographic information system (GIS) software (i.e., ArcGIS Pro 3.0.3) and Python (v. 3.9) scripts. Processing steps per dataset were recorded into the expanding catalog to ensure uniformity and reproducibility of the final product [1].

Guided by the literature review, each of the processed layers were assigned preliminary weights representative of potential socio-economic or environmental cost, or potential risk, associated with a CO_2_ transport routing through said feature. All weights were normalized between zero to one, allowing for direct comparison among layers. In future versions, weights might be adjusted to account for new legislation, guidelines, or stakeholder perspective. Preliminary weighting schemas and reasoning of weighting per dataset were recorded in the data catalog and are included in the metadata that accompanies the CCS Pipeline Route Planning Database [1].

The CCS Pipeline Route Planning Database, metadata file, and documentation are publicly available through the DOE's online data library and research resource, EDX®. This dynamic resource will be updated as new information and legislation are made available.

## Limitations

The majority of original resource datasets spanned all 50 U.S. states, with the exception of the NASA landslide susceptibility dataset, the USGS principal aquifers dataset, the soil frost action potential dataset and the soil steel corrosion dataset. NASA's landslide susceptibility dataset was available for the contiguous U.S. and Alaska, but not Hawaii. The soil frost action potential and soil steel corrosion potential datasets were only available for the contiguous U.S. The USGS principal aquifer dataset was only available for the contiguous U.S. In addition, some resources were available at the county or census tract-level as the finest resolution. These resources include the environmental, energy, and social justice datasets and FEMA's National Risk Index datasets. Due to the size of several original resources, including the nationwide building footprint data, land cover data, high consequence areas data, and floodplains data, custom Python (v. 3.9) scripts were utilized for data handling, which using a windowing method to acquire and process the data in chunks due to the amount and geometric detail of the data provided. Moreover, to make the data more usable for transport-route planning, some datasets were geometrically simplified by spatially dissolving overlapping or touching boundaries, including weighted land cover, building footprints, high consequence areas, and floodplains. Lastly, as noted in the Data Description section, when CO_2_ regulations or legislation were not available, oil and gas regulations were used as reasoning for processing and weighting features.

## Ethics Statements

All authors have read and follow the ethical requirements for publication in Data in Brief and confirm that this work and the data involved does not involve human subjects, animal experiments, or any data collected from social media platforms.

## CRediT authorship contribution statement

**Catherine Schooley:** Conceptualization, Methodology, Data curation, Writing – original draft, Writing – review & editing, Visualization. **Lucy Romeo:** Conceptualization, Methodology, Data curation, Writing – original draft, Writing – review & editing, Visualization, Supervision, Project administration, Funding acquisition. **Isabelle Pfander:** Data curation, Writing – original draft, Writing – review & editing. **Maneesh Sharma:** Data curation, Writing – original draft, Writing – review & editing. **Devin Justman:** Data curation, Writing – original draft, Writing – review & editing. **Jennifer Bauer:** Conceptualization, Supervision, Project administration, Funding acquisition, Writing – review & editing. **Kelly Rose:** Conceptualization, Supervision, Project administration, Funding acquisition, Writing – review & editing.

## Data Availability

CCS Pipeline Routing Database (Reference data) (Energy Data eXchange)
